# Association between rheumatoid arthritis and hyperuricemia among adults: a cross-sectional study based on NHANES data

**DOI:** 10.1007/s10067-025-07386-z

**Published:** 2025-03-06

**Authors:** Chanjing Zhao, Qian Xiao, Wen Huang, Yushun Chen, Xuran Yang

**Affiliations:** 1 Department of Laboratory Medicine, The Second Affiliated Hospital of Guangzhou University of Chinese Medicine, Guangzhou, 510120 Guangdong China; 2 The Second Clinical College of Guangzhou University of Chinese Medicine, Guangzhou, 510006 Guangdong China; 3 Department of Blood Transfusion, Foresea Life Insurance Guangzhou General Hospital, Guangzhou, 511300 Guangdong China; 4 Department of Laboratory Medicine, Xiaolan People’s Hospital of Zhongshan, Xiaolan Town, No.65, Jucheng Avenue, Zhongshan, 528415 Guangdong China

**Keywords:** Arthritis, Cross-sectional study, Hyperuricemia, NHANES, Rheumatoid

## Abstract

**Objectives:**

This study aimed to explore the relationship between rheumatoid arthritis (RA) and hyperuricemia among adults.

**Method:**

All the data were from the National Health and Nutrition Examination Survey (NHANES 1997–2018) database. Linear regression, logistic regression, and restricted cubic spline (RCS) analyses were used to investigate the association between RA and hyperuricemia. Subgroup analysis and interaction tests were conducted to assess the influence of various subgroups on their association.

**Results:**

This study included 41,460 patients, among whom 2603 had RA. The RA group had higher uric acid levels compared with the non-RA group (*P* < 0.001). Linear regression showed that RA was significantly related to uric acid levels among several adjusted models (all *P* < 0.05). Logistic regression analysis also indicated the independent association between RA and hyperuricemia in a positive relationship (*P* < 0.05). Subgroup analysis revealed significant association in the subgroups of females, age ≥ 60 years, non-Hispanics, individuals with hypertension and antihypertensive drugs use, and those with BMI ≥ 30 kg/m^2^ (all *P* < 0.05). The interaction test showed that there was no interaction effect between baseline features and RA (all interaction *P* > 0.05). RCS analysis further found that the course of RA, rather than the age of diagnosis, was related to hyperuricemia (*P* < 0.05). Furthermore, we found that the association between RA and hyperuricemia was mainly observed in populations with 15–30-year course of RA (*P* < 0.05).

**Conclusions:**

RA was associated with hyperuricemia and their association was still stable even after adjusting for several variables, suggesting that uric acid levels should be routinely tested to detect hyperuricemia at an early stage in patients with RA.

**Table:** 

**Key Points**
• *Revealing association between rheumatoid arthritis (RA) and hyperuricemia risk: This study initially explored the association between RA and hyperuricemia, finding that RA was positively related to the higher uric acid levels and hyperuricemia risk*. •* Reflecting the role of RA course on their association: Our study found that their association was mainly observed in population with RA course of 15–30 years*.

## Introduction

Rheumatoid arthritis (RA) is a common chronic inflammatory autoimmune disease characterized by synovial inflammation and proliferation, as well as progressive degradation of cartilage and bone. The prevalence and incidence of RA are both on the rise [1], with a higher prevalence in women, smokers, and those with a family history of RA [2]. Currently, the pathogenesis of RA remains unclear, but it is believed to result from complex interactions among genetic background, environmental factors, and aberrant immune responses [3, 4]. Previous studies have demonstrated the association of systemic immune-inflammation index [5], blood and urinary heavy metals [6], and dietary retinol intake [7] with RA, suggesting their potential utility in predicting the risk of RA. The potential mechanism associated with the RA initiation needs more investigations.

Moreover, current studies suggested that RA was significantly related to the risk of several human diseases. One study reported that RA was associated with an increased likelihood of non-functional dentition [8]. RA also interacted with Mediterranean diet, which increased the risk of cardiovascular disease for the middle aged and elderly [9]. At present, most of the studies focused on the risk factors of RA, but few studies paid attention to the effect of RA to other diseases. Consequently, the influence of RA on other diseases has received limited attention. The characteristic feature of RA is persistent inflammation, and reactive oxygen species are correspondingly increased at the site of inflammation [10]. The various estimation of free radical activity revealed the presence of allantoin in serum or plasma, as the first and major product of uric acid oxidation [11]. This study suggested the abnormal uric acid level. Serum uric acid (SUA) is the final product of purine metabolism, and abnormal metabolism can lead to hyperuricemia. Hyperuricemia is the result of interactions among various factors, including gender, age, genetics, lifestyle, and environmental factors. Epidemiological data have shown that the prevalence of hyperuricemia in the USA has steadily increased from the 1960s to the 1990s and has remained stable from 2007 to 2016, and also showed significant escalating trends in Chinese adults [12, 13]. However, no study revealed the association between RA and hyperuricemia.

In this study, we analyzed the data from the NHANES database spanning from 1999 to 2018 to determine the prevalence of hyperuricemia in RA patients and the general population, and clarified the association between RA and hyperuricemia by 3 regression models. We also explored the relationship of diagnosis age of RA and RA course on the risk of hyperuricemia. This study highlights the potential role of RA in the development of hyperuricemia.

## Methods

### Data selection and study design

The research design of this study was based on a cross-sectional study design, and the data analyzed were obtained from the National Health and Nutrition Examination Survey (NHANES). The database includes demographic data, dietary data, examination data, laboratory data, questionnaire data, and limited access data. NHANES was conducted over 10 cycles from 1999 to 2018. This research received approval from the National Center for Health Statistics Research Ethics Review Board, and participants signed informed consent forms. The detailed NHANES study design and data are publicly available at https://www.cdc.gov/nchs/nhanes/.

**Fig. 1:**
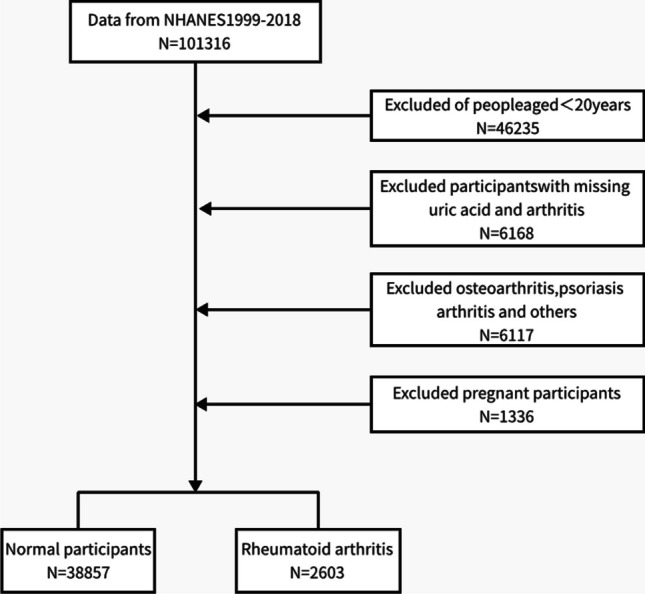
Flowchart of the participants selection from NHANES 1999–2018

The exclusion criteria for this study were as follows: (1) age < 20 years; (2) pregnant individuals; (3) individuals lacking arthritis data; (4) individuals lacking serum uric acid data. The flow chart of this study is presented in Fig. 1.

Ultimately, 41,460 individuals were included. In this study, the data were weighted according to the sample weight calculation method recommended by NHANES. We chose Mobile Examination Centers (MEC) weights. The weight calculation formula for 1999–2000 and 2001–2002 was 2/10 × wtmec4yr, and the weight calculation formula for 2003–2018 was 1/10 × wtmec2yr.

### Definitions of rheumatoid arthritis and hyperuricemia

The definition of RA was obtained through self-reported questionnaires. Participants were first asked, “Did a doctor or other healthcare professional ever tell you that you have arthritis?” Those who answered “no” were included in the non-RA group. Those who answered “yes” proceeded to the second question, “What type of arthritis was it?” Participants who answered “rheumatoid arthritis” were included in the RA group, while those who answered differently were included in the non-RA group [14]. We also obtained the information of age when told that patients had arthritis (age of diagnosis). The course of RA can be known by the current age minus the age of diagnosis.

The definition of hyperuricemia was based on serum uric acid levels, with levels exceeding 7 mg/dL in males and 6 mg/dL in females classified as hyperuricemia [15].

### Assessment of covariates

The covariates included in this study are gender, age (years), race, BMI (Body Mass Index, kg/m^2^), education level, Poverty Income Ratio (PIR), alcohol history, hypertension, diabetes, cardiovascular disease, albumin (g/L), glomerular filtration rate (GFR, ml/min), HDL-cholesterol (HDLC, mg/dL), LDL-cholesterol (LDLC, mg/dL), total cholesterol (TC, mg/dL), triglyceride (TG, mg/dL), and antihypertensive drugs use. Hypertension is defined as a physician’s diagnosis, taking antihypertensive medications, SBP/DBP ≥ 140/90 mmHg; diabetes mellitus is defined as a physician’s diagnosis, taking antidiabetic drugs, HbA1c ≥ 6.5%, FPG ≥ 7.0 mmol/L. Cardiovascular diseases were defined as a positive answer to the question “Have you ever been told you had congestive heart failure/coronary heart disease/angina/heart attack/stroke?”.

### Statistical methods

All analyses were performed using SPSS and R (version 4.1.3). The NHANES database was surveyed using complex, multi-stage, sampling; therefore, our study used MEC exam weight (WTMEC4YR, WTMEC2YR) for analysis. Continuous variables are presented as weighted means and standard deviations or median, and categorical variables are presented as weighted percentages. We compared categorical variables and continuous variables between different groups using the chi-square test and T-test, respectively. We employed generalized linear regression and logistic regression analyses to analyze the association between RA and hyperuricemia. Stratifying and conducting interaction analyses were also performed. In addition, the Restricted cubic spline (RCS) was used to assess the correlation between 2 variables. All statistical tests were two-sided, and a *P* < 0.05 was statistically significant.

## Results

### General characteristics of the study population

A total of 41,460 participants were included in this study, of which 2603 (6.28%) had rheumatoid arthritis (RA). The prevalence of hyperuricemia in RA patients was 27.40%, compared to 18.18% in the general population.

The clinical characteristics of participants are presented in Table 1. Compared to the non-RA group, patients with RA were more likely to be female, older, non-Hispanic Black. RA group have lower educational attainment, PIR (Poverty Income Ratio), have higher BMI, albumin level, TG level, and more likely to have alcohol consumers, hypertension, diabetes, cardiovascular disease, hyperuricemia and antihypertensive drugs use (*P* < 0.05). There were no differences between the 2 groups in terms of HDLC, LDLC, and TC levels.

**Table 1:** Weighted characteristics of the population

Characteristic	RA(*N* = 2603)	Non-RA(*N* = 38857)	*P*
Gender, *n* (%)			
Male	40.93	51.32	< 0.001
Female	59.07	48.68	
Age (years)	58.01 ± 14.47	44.68 ± 16.23	< 0.001
Race, *n* (%)			< 0.001
Mexican American	6.51	9.06	
Other Hispanic	4.86	6.11	
Non-Hispanic White	67.08	66.69	
Non-Hispanic Black	16.08	10.97	
Other Race	5.47	7.17	
Education, *n* (%)			< 0.001
Less than high school	26.56	17.07	
High school	28.24	23.82	
More than high school	45.21	59.11	
PIR	2.50 ± 1.59	3.02 ± 1.64	< 0.001
BMI (kg/m^2^)	30.27 ± 7.37	28.37 ± 6.46	
Drink, *n* (%)			0.031
Yes	56.33	51.66	
No	43.67	48.34	
Hypertension, *n* (%)			< 0.001
Yes	61.62	49.39	
No	38.38	67.03	
Diabetes, *n* (%)			< 0.001
Yes	23.03	10.37	
No	76.97	89.63	
CVD, *n* (%)			< 0.001
Yes	24.19	6.42	
No	75.81	93.58	
Albumin	9.9 (4.9, 24.6)	8.0 (4.2, 16.4)	< 0.001
GFR	75.81 (55.81, 103.18)	83.41 (61.89, 111.40)	< 0.001
HDLC	50 (41, 62)	50 (41, 61)	0.536
LDLC	113 (89, 137)	113 (91, 137)	0.281
TC	193 (166, 222)	192 (166, 220)	0.151
TG	115 (82, 170)	105 (72, 155)	< 0.001
Uric acid	5.61 ± 1.61	5.40 ± 1.40	< 0.001
Hyperuricemia, *n* (%)			
Yes	27.40	18.18	< 0.001
No	72.60	81.82	
Antihypertensive drugs			
Yes	1307	8700	< 0.001
No	104	1727	

### Relationship between RA and hyperuricemia

We first explored the association between RA and uric acid levels by generalized linear regression after adjusting for different variables (Table 2). The results showed that RA was positively associated with uric acid levels in crude model (β = 0.236, 95%CI: 0.178–0.293, *P* < 0.001). Their association was still significant in adjusted model 1 (β = 0.103, 95%CI: 0.051–0.156, *P* < 0.001) and model 3 (β = 0.278, 95%CI: 0.202–0.354, *P* < 0.001). However, no association was observed between them after adjusting for disease history and antihypertensive drugs use, which suggested the potential role of the antihypertensive drugs use on their association.

**Table 2:** Relationship between RA and hyperuricemia

	Uric acid levels (β, 95%CI)	Hyperuricemia (OR, 95%CI)
Crude Model	0.236 (0.178, 0.293), *P* < 0.001	1.721 (1.576, 1.881), *P* < 0.001
Model 1	0.103 (0.051, 0.156), *P* < 0.001	1.180 (1.066, 1.307), *P* = 0.001
Model 2	0.139 (− 0.009, 0.288), *P* = 0.066	1.357 (1.107, 1.664), *P* = 0.003
Model 3	0.278 (0.202, 0.354), *P* < 0.001	1.853 (1.615, 2.126), *P* < 0.001

Crude model: No variable was adjusted

Model 1: Adjusted for sex, age, race, BMI, education level, and PIR

Model 2: Adjusted for alcohol, hypertension, diabetes, cardiovascular diseases, and antihypertensive drugs use

Model 3: Adjusted for albumin, HDLC, LDLC, TC, TG, and GFR

We then explored the association between RA and hyperuricemia by logistic regression analyses, and all models indicated a positive association between them (crude model, OR = 1.721, 95%CI: 1.576–1.881, *P* < 0.001; model 1, OR = 1.180, 9%CI: 1.066–1.307, *P* = 0.001; model 2, OR = 1.357, 95%CI: 1.107–1.664, *P* = 0.003; model 3, OR = 1.853, 95%CI: 1.615–2.126, *P* < 0.001). The risk of developing hyperuricemia was significantly higher in patients with RA.

We also performed a subgroup analysis to verify the stability of their association. The subgroup analysis results showed inconsistent associations between hyperuricemia and RA across different subgroups (Table 3). Statistically significant associations (*P* < 0.05) were observed in the subgroups of females, age ≥ 60 years, non-Hispanics, individuals with hypertension, individuals with antihypertensive drugs use, and those with BMI ≥ 30 kg/m^2^. Additionally, interaction tests indicated that gender, age, race, alcohol consumption, hypertension, diabetes, cardiovascular disease, antihypertensive drugs use, and BMI did not significantly modify this relationship (interaction all *P* > 0.05).

**Table 3:** Subgroup analysis of the association between RA and hyperuricemia

Subgroup	OR (95%CI)	*P* for interaction
Gender		0.930
Male	1.173 (0.785,1.752), *P* = 0.437	
Female	0.170 (0.055,0.524), *P* = 0.002	
Age (years)		0.745
< 60	1.110(0.777, 1.588), *P* = 0.566	
≥ 60	2.416(1.093, 5.339), *P* = 0.029	
Race		0.560
Mexican American	0.882 (0.522, 1.491), *P* = 0.639	
Other Hispanic	0.047 (0.003, 0.666), *P* = 0.024	
Non-Hispanic White	3.562 (0.731, 17.361), *P* = 0.116	
Non-Hispanic Black	1.274 (0.236, 6.895), *P* = 0.778	
Other Race	3.062 (0.311, 30.167), *P* = 0.338	
Hypertension		0.150
Yes	1.280 (1.038, 1.578), *P* = 0.021	
No	0.718 (0.258, 1.994), *P* = 0.525	
Diabetes		0.655
Yes	1.243 (0.923, 1.675), *P* = 0.153	
No	1.409 (0.393, 5.049), *P* = 0.599	
Drink		0.802
Yes	1.234(0.951, 1.601), *P* = 0.114	
No	0.805(0.276, 2.350), *P* = 0.691	
CVD		0.210
Yes	0.983 (0.681, 1.419), *P* = 0.926	
No	2.936 (0.559, 15.406), *P* = 0.203	
BMI		0.188
< 25	1.292 (0.764, 2.184), *P* = 0.338	
25–30	1.678 (0.444, 6.341), *P* = 0.445	
≥ 30	5.910 (1.790, 19.512), *P* = 0.003	
Antihypertensive drugs		
Yes	1.185 (1.050, 1.338), *P* = 0.006	0.300
No	1.505 (0.974, 2.326), *P* = 0.066	

### Role of RA course on their relationship

We finally explored the influence of diagnosis age of RA (when told participants had RA) and the course of RA on hyperuricemia. Because the data of diagnosis age (when told participants had RA) was only recorded in the 2007–2018 year; therefore, the analysis in this section was only conducted on the data from 2007 to 2018 year.

**Fig. 2:**
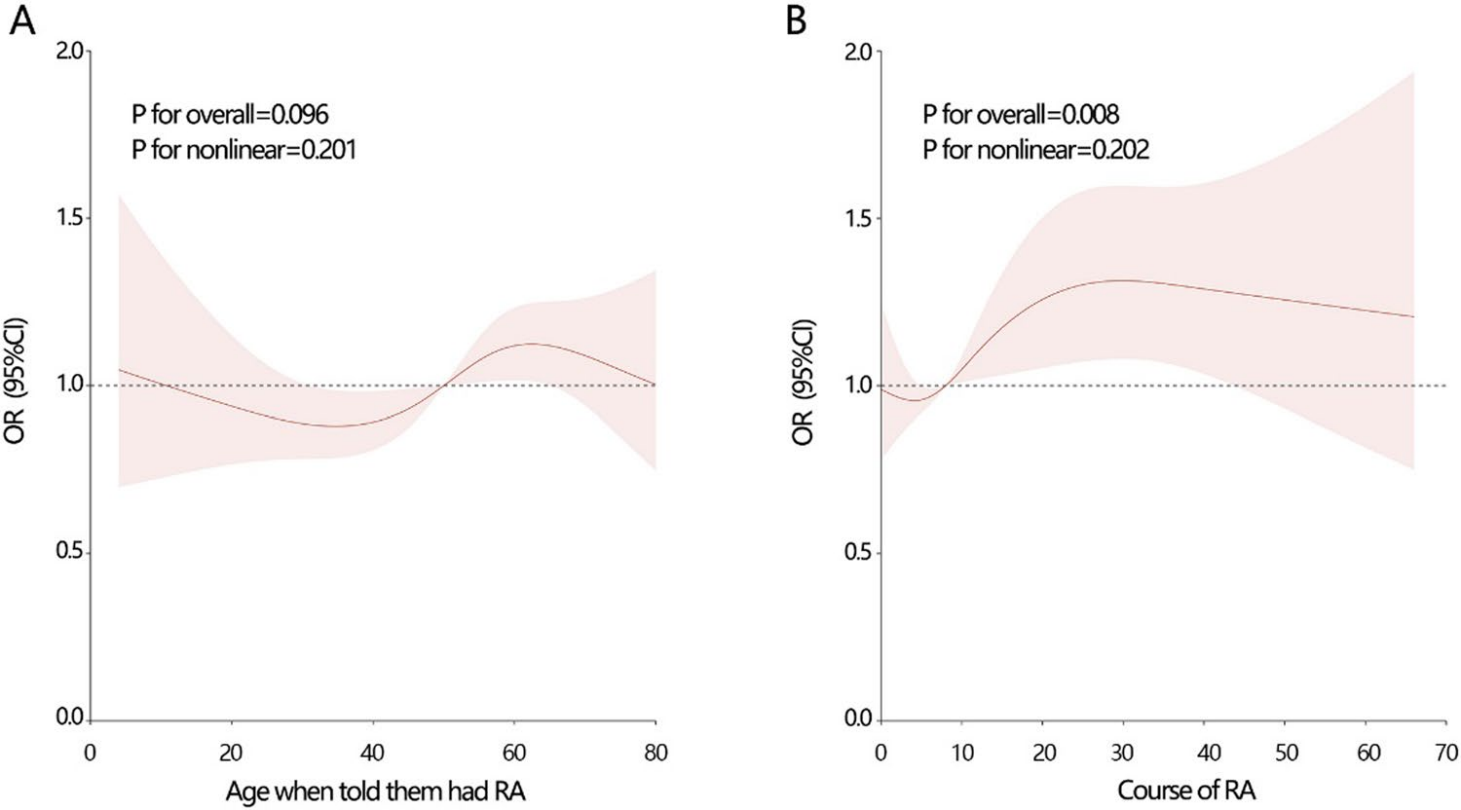
The correlation analysis by RCS analysis. **A** Between age of diagnosis (when told participants had RA) and hyperuricemia. **B** Between the course of RA and hyperuricemia. The course of RA can be known by current age minus the age when told patients had arthritis

The RCS analysis indicated that diagnosis age of RA had no significant correlation with hyperuricemia risk (Fig. 2A, P for overall = 0.096). However, the course of RA showed a significant correlation with hyperuricemia (Fig. 2B, P for overall = 0.008).

Considering the potential influence of the course of RA on the association between RA and hyperuricemia, we further explored their association among populations with different course of RA (Table 4). The results showed that their association was mainly observed in the population with RA course of 15–30 years (*P* = 0.032 in crude model; *P* = 0.004 in model 3). No association was found between them among all models in populations with RA course less than 15 or greater than 30 years (all *P* > 0.05). Our results initially highlighted the importance of RA course on their association.

**Table 4:** Relationship between RA and hyperuricemia stratified by the course of RA

	< 15 years	15–30 years	> 30 years
Crude model	0.983 [0.827, 1.169] *P* = 0.849	1.362 [1.027, 1.806] *P* = 0.032	0.821 [0.521, 1.294] *P* = 0.395
Model 1	0.968 [0.799,1.172] *P* = 0.739	1.225 [0.887, 1.692] *P* = 0.217	0.852 [0.501, 1.447] *P* = 0.553
Model 2	1.384 [0.896, 2.137] *P* = 0.143	1.073 [0.536, 2.148] *P* = 0.843	1.180 [0.387, 3.596] *P* = 0.770
Model 3	0.993 [0.766, 1.287] *P* = 0.957	1.914 [1.232, 2.974] *P* = 0.004	1.282 [0.597, 2.755] *P* = 0.524

Crude model: No variable was adjusted

Model 1: Adjusted for sex, age, race, BMI, education level, and PIR

Model 2: Adjusted for alcohol, hypertension, diabetes, cardiovascular diseases, and antihypertensive drugs use

Model 3: Adjusted for albumin, HDLC, LDLC, TC, TG, and GFR

## Discussion

This study utilized data from the NHANES database spanning from 1999 to 2018 to investigate the association between RA and hyperuricemia. Based on estimates from NHANES data between 2007 and 2016, the prevalence of hyperuricemia was 18.38% [16]. Our study reported a prevalence of 27.40% for hyperuricemia among RA patients, and further analysis revealed that RA was associated with hyperuricemia. These findings suggested that RA patients should undergo regular screening of uric acid levels to effectively manage patient prognosis.

This study found that uric acid levels in patients with RA were significantly higher compared to the general population. One study reported that 15% of people with RA were in high uric acid level, with an average of 409 ± 44.4 μmol/L [17]. A study from Egypt also suggested that elevated uric acid levels were common among RA patients and may serve as a marker of inflammation severity in joints [18]. However, other studies have shown that uric acid concentrations in RA patients were similar to those in healthy populations [11]. It followed that the association between RA and uric acid levels was uncertain and needed more investigations. The mechanism of the association between RA and uric acid levels is complex. RA patients frequently exhibited disturbances in purine metabolism due to chronic inflammation, which accelerated cell death and the subsequent release of nucleic acids, ultimately leading to elevated uric acid levels [19]. Therefore, in patients with RA, serum uric acid levels should be routinely tested to detect hyperuricemia at an early stage. Recent study also has elucidated that uric acid plays a pivotal role in driving inflammation by activating fibroblast-like synoviocytes (FLS) through intracellular signal transduction mechanisms in RA [20]. Uric acid crystals, as well as soluble uric acid, have been shown to activate the NLRP3 inflammasome, resulting in the secretion of pro-inflammatory cytokines such as IL-1β and IL-18, which significantly contribute to the exacerbation of inflammatory responses in RA [21, 22]. Additionally, uric acid has been implicated in promoting the differentiation of Th17 cells while suppressing the function of regulatory T cells, thereby disrupting immune homeostasis and potentially driving RA pathogenesis [23]. These knowledges suggested that hyperuricemia may exacerbate the symptoms of RA by promoting inflammatory responses and oxidative stress. Therefore, effective anti-inflammatory treatment is particularly important. Actively managing uric acid levels can help reduce the risk of joint damage, especially in elderly patients or those with multiple comorbidities.

Owing to the chronic inflammatory nature, patients with RA have a higher risk of cardiovascular disease compared to the general population, where cardiovascular disease is the most common cause of mortality. Therefore, optimizing the prognosis of RA patients is crucial in their management. In the general population, cardiovascular disease can be prevented through established screening methods and interventions aimed at reducing risk. However, the outcomes differ in RA patients. The study found that when patients with RA were treated with methotrexate, a disease-modifying antirheumatic drug, the cardiovascular morbidity and mortality were significantly reduced as methotrexate had the lowering effect on arterial blood pressure [24]. The previous study suggested that risk assessment focusing on the prevention of RA will become a public health strategy, just like managing cardiovascular diseases today [25]. But a study based on three continents found that although RA patients used drugs such as statins, CVD risk were usually not fully controlled [26]. This may be because the RA was an independent cardiovascular risk factor although RA was associated with an increased risk of cardiovascular disease and mortality [27]. These findings suggested that the strategies to improve the prognosis of RA patients were not enough to focus only on risk factors of cardiovascular disease.

This study revealed a significant association between rheumatoid arthritis and hyperuricemia. It is important to note that hyperuricemia was associated with many diseases. It has been found that elevated SUA levels were related to the low bone mineral density in pre- but not post-menopausal women with RA [28] SUA levels were positively related to carotid intima-media thickness (IMT). Hyperuricemia increased the risk of atherosclerosis by 50%, and gut microbiota can affect these chronic diseases [29, 30]. In addition, SUA levels were also involved in the blood pressure [31], hypertriglyceridemia [32], periodontitis [33], muscle strength [34], and psoriasis [35]. These findings suggest that monitoring SUA levels may contribute to reducing the risk of several diseases and improve the quality of life.

Our research highlighted that screening uric acid levels is a crucial management measure for patients with RA. Apart from preventing hyperuricemia, it can also help in the prevention of cardiovascular disease, metabolic syndrome, and chronic kidney disease. However, several limitations should also be stated. The NHANES database did not provide the data about uric acid lowering drugs usage, therefore the effect of uric acid lowering drugs was not considered. In addition, RA was self-reported by patients in NHAENS, therefore their current situation cannot be evaluated.

## Conclusion

In summary, our study found a significant association between RA and hyperuricemia, and their association was stable even after adjusting for several variables. Our study suggested that in patients with RA, uric acid levels should be routinely tested to detect hyperuricemia at an early stage. As this study was based on a cross-sectional design, it was unable to establish the temporal sequence or infer a causal relationship between RA and hyperuricemia. Therefore, future studies, particularly longitudinal studies, were essential to further explore and clarify the potential causal mechanisms between them.

## Data Availability

The datasets generated during and/or analyzed during the current study are available from the corresponding author on reasonable request.
